# Treatment of Chrysanthemum Synthetic Seeds by Air SDBD Plasma

**DOI:** 10.3390/plants11070907

**Published:** 2022-03-29

**Authors:** Nikola Škoro, Suzana Živković, Slađana Jevremović, Nevena Puač

**Affiliations:** 1 Institute of Physics—National Institute of Republic of Serbia, University of Belgrade, Pregrevica 118, 11080 Belgrade, Serbia; nevena@ipb.ac.rs; 2 Institute for Biological Research “Siniša Stanković”—National Institute of Republic of Serbia, University of Belgrade, Despot Stefan Boulevard 142, 11000 Belgrade, Serbia; suzy@ibiss.bg.ac.rs

**Keywords:** artificial seeds, plasma treatment, chrysanthemum, dielectric barrier discharge, cold plasma

## Abstract

Herein, we present the effect of surface dielectric barrier discharge (SDBD) air cold plasma on regrowth of chrysanthemum synthetic seeds (synseeds) and subsequent plantlet development. The plasma system used in this study operates in air at the frequency of 50 Hz. The detailed electrical characterization of SDBD was shown, as well as air plasma emission spectra obtained by optical emission spectroscopy. The chrysanthemum synseeds (encapsulated shoot tips) were treated in air plasma for different treatment times (0, 5 or 10 min). Plasma treatment significantly improved the regrowth and whole plantlet development of chrysanthemum synseeds under aseptic (in vitro) and non-aseptic (ex vitro) conditions. We evaluated the effect of SDBD plasma on synseed germination of four chrysanthemum cultivars after direct sowing in soil. Germination of synseeds directly sowed in soil was cultivar-dependent and 1.6−3.7 fold higher after plasma treatment in comparison with untreated synseeds. The study showed a highly effective novel strategy for direct conversion of simple monolayer alginate chrysanthemum synseeds into entire plantlets by plasma pre-conversion treatment. This treatment reduced contamination and displayed a considerable ex vitro ability to convert clonally identical chrysanthemum plants.

## 1. Introduction

Synthetic seed technology is one of the most promising tools in plant biotechnology and may represent an innovative method for massive plant production and sustainable agriculture in the future [1]. Synthetic seeds (artificial seeds or synseeds) have been defined as artificially encapsulated somatic embryos or other non-embryogenic vegetative parts of plants, mainly in alginate, that may be used for storage or sowing under in vitro or ex vitro conditions [2]. The term ‘synseeds’ was described by Murashige in 1977 [3] as ‘an encapsulated single somatic embryo’, but later, the definition of artificial seeds was extended to any artificially coated micropropagules that have capability to be sown as a seed and converted into a plant [4,5]. There is a growing trend in applications of synseed technology for medium- and long-term storage of plant material under aseptic conditions [6,7] or as an advanced procedure of cryopreservation by encapsulation–dehydration and encapsulation–vitrification method [8,9]. Synseed technology represents an efficient alternative technique for propagation and germplasm conservation of valuable forest, medicinal and vegetable plant species that reproduce mainly vegetatively or have a problem in seed propagation, i.e., plants that produce non-viable seeds or seedless plants [10].

In synseed technology, an alginate capsule has two roles: (i) it acts as physical barrier of shoot tips against mechanical damage, and (ii) it serves as an artificial endosperm, carbon source and reservoir of nutrients for better survival and supply of energy [9]. Alginates are a group of naturally occurring anionic polysaccharides derived from brown algae cell walls (*Macrocystis pyrifera*, *Limnaris hyperborea*, *Ascophyllum nodosum*) and several bacterial strains (*Azotobacter*, *Pseudomonas*). Sodium alginate is soluble in water, but when the sodium is replaced with calcium, the ionic bond with calcium cross links the polymer chain in alginate, which results in the formation of an insoluble gel. Sodium alginate and calcium salt are reported to be the best combination for encapsulation, representing the most successful and widely accepted approach to synseed production [9]. Alginates can be formed into diverse semisolid or solid structures because of their ability of sol/gel transition and are commonly used as viscosity-increasing agents, thickeners and suspension and emulsion stabilizers in food and the pharmaceutical industry (code E400-E405) [11]. In addition, alginate gels are the basis for a variety of wound dressings that have showed variety of therapeutically effects, such as hemostatic and bacteriostatic properties [12,13]. On the other hand, in plants, sodium alginate is considered a potential elicitor that improves tolerance to plant environmental stresses, such as drought, inhibiting plant infections and reducing the toxic effect of heavy metals [14,15].

Chrysanthemums (*Chrysanthemum morifolium* Ramat. syn. *C. grandiflorum* Kitam) are, besides roses, the most important economically ornamental crop in the world [16]. They originate from east Asia, a center of their biodiversity; however, to date, many horticultural varieties and cultivars of chrysanthemums are produced using different biotechnological tools [17]. The name chrysanthemum means gold flower, but they are also called “autumn roses” because they were, in the past, used as cut flowers during late summer and autumn. Nowadays, there is constant demand on the market for new cultivars that are available during the whole year. Modern biotechnological tools, such as mutation breeding and micropropagation under in vitro conditions, allow for production of hundreds of new chrysanthemum cultivars every year [18]. Chrysanthemum cultivars are commonly propagated vegetatively by cuttings and suckers and stored as field, greenhouse or in vitro collections due to high spontaneous mutation rates and high levels of ploidy and self-incompatibility [19,20]. Micropropagation of chrysanthemum cultivars, as an in vitro way of vegetative multiplication in culture, was reported for the first time more than 50 years ago [21], and numerous reports about plantlet regeneration from various explants of chrysanthemum have been presented [16,22,23,24].

The application of synseed technology, accompanied by micropropagation, represents a perfect biotechnological approach that could be used for agricultural improvement of year-round plant production of chrysanthemums. There are several advantages of this approach, including large-scale production; easy handling; short and medium storage (4 °C) or low temperature (−196 °C) storage; easy transportation; and the genetically true-to-type nature of the plants produced from synseeds. On the other hand, there are some limitations of wider usage of synseed technology in commercial applications as published to date, such as implementation of labor-intensive procedures, which include double-layer encapsulation or several media changes to derived plantlets with well-developed shoot and roots at the same time. To date, the application of synseed technology of chrysanthemum cultivars has been investigated for in vitro storage and ex vitro planting (summarized in Table 1). In addition, synseed technology in chrysanthemums is widely used as a part of encapsulation–dehydration and encapsulation–vitrification protocols for long-term storage of chrysanthemum cultivars by cryopreservation in liquid nitrogen [25]. Considering the fact that chrysanthemums are susceptible to mutations, meristem explants (i.e., nodal segments or shoot tips) proved to be the best explant choice for the plant propagation of chrysanthemums, with a high degree of clonal fidelity as mother plants, as well as for synseed production [26,27,28].

Chrysanthemum synseeds are mainly produced under sterile conditions for short- and long-term storage [19], but sowing of chrysanthemum synthetic seeds under non-aseptic conditions has been also reported [29,30]. Chrysanthemum synseeds easily regrow from Na-alginate beads under sterile conditions, whereas for complete germination and whole-plantlet development (shoot and root), it is necessary to add indole-acetic acid to the encapsulation matrix [29] or, as separate step, in the medium for rooting after shoot regrowth [19]. For sowing under unsterile conditions, the results showed that presence of organic compounds in the gelling matrix and commercial substrates caused microbial contamination in all synseeds and complete inhibition of further regrowth of the shoots or whole plantlet development. In general, difficulties of sowing artificial seeds directly in soil or in commercial substrates, such as compost, vermiculite, etc., under non-sterile conditions are considered to be one of the main limitations for the widescale practical application of synseed technology [2,31]. Some progress has been achieved by using chemical mixtures and antibiotics for preservation of synseeds before sowing [32], but more investigations and novel approaches are still needed to improve the capacity of synseed cultivation under non-sterile conditions.

Atmospheric pressure plasma (non-thermal, “cold”) systems have been extensively used in biomedical applications for almost two decades [33,34,35,36]. In parallel, another field of plasma applications has been growing-plasma agriculture [37,38]. One of the first applications of cold plasmas was the treatments of conventional seeds [39,40]. This includes various applications in seed treatment with several purposes [41,42,43]. Many authors have shown that plasma treatments can increase seed germination and speed up the whole process of plantlet development [37,38,39,40]. The rich plasma chemistry (with reactive oxygen and nitrogen species) interacts with the seed coating and triggers various responses, such as increasing water uptake, changes in the surface of seeds’ coating and elimination of pathogens on seed surfaces [44,45,46,47,48,49,50,51,52,53]. In this sense, cold plasma treatment can have a multiply positive impact on seed germination and subsequent plant development of conventional seeds without the addition of chemicals that can be harmful for the environment.

Nowadays, there is a plethora of plasma sources that operate at atmospheric pressure [54,55,56,57]. They differ in electrode design, type of applied voltage, feeding gas, etc. The variety of atmospheric pressure plasma sources enables a large number of possible applications, but at the same time, comparison between the results of treatment is difficult. Therefore, it is of the utmost importance to obtain detailed characteristics of the plasma device that is used in experiments. One of the first steps that is usually performed includes electrical characterization of the discharge, accompanied by optical emission spectroscopy, which can give insight into the plasma-excited species. These diagnostic techniques represent only the starting point for a detailed description of plasma characteristics and include mass spectrometry, fast imaging, laser-induced fluorescence, etc. [56].

In this paper, we present results of air plasma treatment of *Chrysanthemum* synseeds together with detailed plasma source diagnostics. We performed electrical characterization of the optical emission spectra (OES) of an SDBD system that operates in air at atmospheric pressure. Until now, we are not aware of any research data in the literature about the effect of cold atmospheric plasma treatment on the regrowth (germination) of artificial seeds of any plant species. The objective of this study related to synseed treatment was to: (1) investigate the influence of cold plasma treatment on chrysanthemum synseed regrowth under in vitro conditions; (2) analyze the impact of cold plasma treatment on regrowth and further plantlet development from synseeds sowed directly in soil (ex vitro); and (3) evaluate the effect of plasma treatment on synseed germination of different chrysanthemum cultivars. In this pioneer work, we present a novel approach in synseed biotechnology and plasma agriculture cold plasma treatment of synseeds (encapsulated shoot tips) prior to sowing to prevent contamination and enhance plant growth.

## 2. Results

### 2.1. Regrowth of Plasma-Treated Synthetic Seeds under Aseptic Conditions (In Vitro)

**Figure 1:**
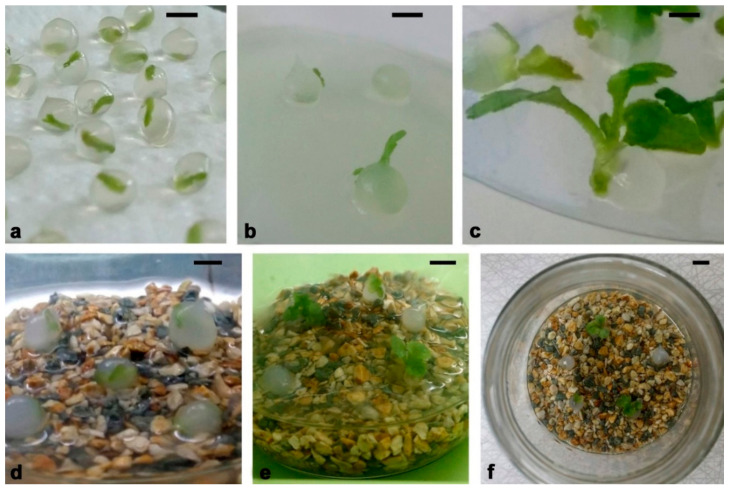
Chrysanthemum synseed germination under in vitro conditions. (**a**) Synseeds before plasma treatment; (**b**,**c**) plasma-treated synseeds grown on solid agar medium—leaf emergence after one week of culture (**b**) and shoot development after four weeks of culture (**c**); (**d**,**f**) plasma-treated synseeds grown on vermiculite + liquid medium—plasma-treated synseeds grown one week (**d**) and four weeks (**e**,**f**). Bars a–f, 5 mm.

**Figure 2:**
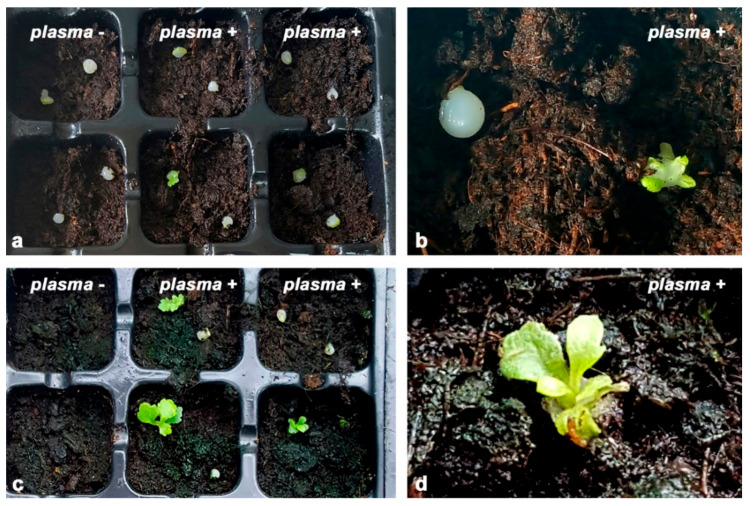
Chrysanthemum synseed germination under ex vitro conditions, cv. Précocita carna. (**a**) Control (first column) and plasma-treated synseeds (second and third column) after one week of growth; (**b**) first sign of germination (leaf emergence) of plasma-treated synseeds; (**c**) control (first column) and plasma-treated synseeds (second and third column) after three weeks of cultivation ex vitro; (**d**) detail of fully germinated plasma-treated chrysanthemum synseed four weeks after sowing.

**Figure 3:**
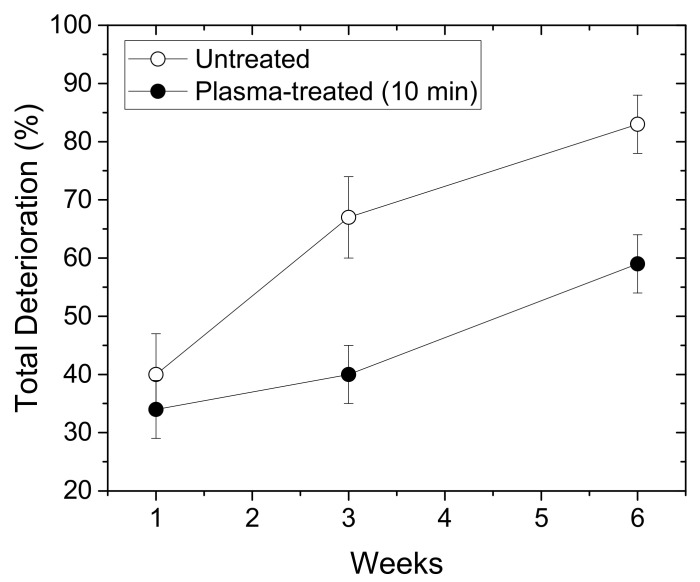
Total deterioration (%) of chrysanthemum synseeds grown ex vitro during 6-week period. Empty symbols: untreated synseeds; full symbols: plasma-treated synseeds.

**Figure 4:**
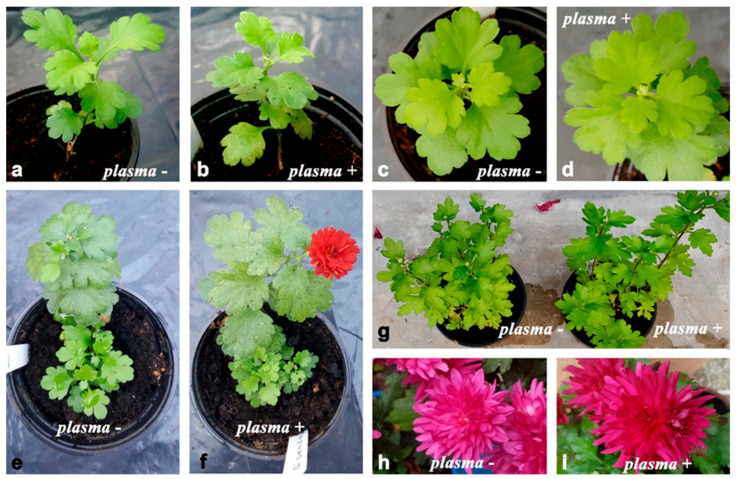
Chrysanthemum plantlets derived from synseeds during growth under greenhouse conditions (ex vitro). (**a***–***d**) Plants derived from untreated (**a**,**c**) and plasma-treated (**b**,**d**) synseeds of chrysanthemum cv. PC (**a**,**b**) and Q (**c**,**d**) three months after sowing; (**e**,**f**) Plantlets derived from untreated (**e**) and plasma-treated (**f**) synseeds of cv. PC six months after sowing; (**g***–***i**) untreated (left) and plasma-treated (right) chrysanthemum plants twelve months after sowing, during next flowering season, cv. BL; (**h**,**i**) flowering of chrysanthemum plants derived from untreated (**h**) and plasma-treated synseeds (**i**), cv. BL.

We tested the regrowth rate of chrysanthemum synseeds after plasma treatment cultivated under in vitro (Figure 1) and ex vitro conditions (Figure 2, Figure 3 and Figure 4). The regrowth and shoot development of plasma-treated and untreated chrysanthemum monolayered, simple synseeds were evaluated for two growing substrates without plant growth regulators under aseptic (in vitro) conditions (Figure 1).

After plasma treatment, one set of synseeds was grown on the solid agar medium (AM, Figure 1b,c), and the second set was placed on the vermiculite + liquid medium (VLM, Figure 1d–f). Untreated synseeds served as a control group and were grown on the same medium. Encapsulated chrysanthemum shoot tips cultivated on AM in vitro (Figure 1a) easily continued to grow after plasma treatment, and within the first week, leaf emergence was observed (Figure 1b). Full development of microshoots was established after four weeks of culture (Figure 1c). There was no apparent difference in regrowth between the control (untreated) and plasma-treated synseeds. Additionally, no contamination was observed among treated synseeds. All synseeds, including the control as well as plasma-treated synseeds, were fully developed in microshoots. We further evaluated shoot multiplication after plasma treatment, and no difference between plasma-treated and untreated synseeds was observed (Supplementary Data, Figure S1).

When chrysanthemum synseeds were grown on VLM (sterilized vermiculite moisture with liquid plant regulator free medium) under in vitro conditions (Figure 1d–f), a significant increase in regrowth of plasma-treated synseeds was achieved (Table 2).

After one week of culture, only 20% of untreated synseeds broke out of the alginate capsule, and none of them continued their growth. On the other hand, among plasma-treated synseeds, leaf emergence, as a first sign of synseed germination, was two- to three-fold higher in comparison to untreated synseeds after one week of culture. The best results for shoot regrowth, more than three times higher than that of untreated synseeds, was achieved after plasma treatment for 5 min and 10 min before planting in VLM. After four weeks of cultivation, only plasma-treated chrysanthemum synseeds (33–47%) continued their growth and developed microshoots (Figure 1e,f), whereas the untreated (control) synseeds did not survive on VLM. For further experiments on chrysanthemum synseeds, we used 10 min plasma treatment.

### 2.2. Germination of Plasma-Treated Synthetic Seeds under Non-Aseptic Conditions (Ex Vitro)

We examined the germination of plasma-treated and untreated chrysanthemum synseeds, as well as the subsequent growth and development of plantlets after direct sowing in soil substrate (ex vitro conditions) (Table 3, Figure 2). Plasma-treated synseeds showed more vigorous survival, regrowth and germination in comparison to untreated synseeds (Table 3). After one week of ex vitro cultivation, shoots broke the Na-alginate bead, and the first leaf emerged from the synseeds (Figure 2a,b), which represented the main sign of shoot regrowth. The difference in survival between plasma-untreated (first column) and plasma-treated synseeds (second and third column) was evident (Figure 2a,c). Despite the fact that there was no statistically significant difference in shoot regrowth of plasma-treated (66%) and untreated synseeds (60%), after one week of growth under ex vitro conditions, we noticed that the developed leaves formed from plasma-treated synseeds were wider than those derived from untreated synseeds (Figure 2a). After one week of growth, some untreated synseeds were lost due to desiccation and contamination.

After three weeks of growing under ex vitro conditions, we observed two times higher survival and almost doubled percentage of shoot development of plasma-treated chrysanthemum synseeds in comparison to untreated samples. Namely, only 33.3% of untreated synseeds continued growth and developed very well-formed shoots, whereas more than 60% of plasma-treated chrysanthemum synseeds formed well-developed shoots (Figure 2c). Six weeks after sowing, whole chrysanthemum plantlets with well-formed shoots and roots were developed (Table 3). Only 17% of untreated synseeds fully germinated and developed whole plantlets, whereas 2.5 fold higher synseed germination (over 40%) and whole-plantlet development of plasma-treated synseeds was achieved.

We compared the total deterioration (%) of the chrysanthemum plasma-treated and untreated synseeds within the first 6 weeks of growth ex vitro (Figure 3). The values reached almost 85% in the case of untreated synseeds, whereas for the plasma-treated seeds, this percentage was around 60%. According to these results, we can conclude that the plasma treatment of synseeds before sowing under ex vitro conditions enables increased survival of the newly developed plants by 25%.

### 2.3. Germination of Plasma-Treated Synthetic Seeds of Different Cultivars Ex Vitro

We evaluated the effect of plasma treatment (10 min) on synseed germination of different chrysanthemum cultivars (Table 4, Figure 4). We found that the plasma treatment significantly enhanced the process of synseed germination and conversion to plantlet after direct sowing in soil for all tested cultivars. This effect of plasma was cultivar-dependent (Table 4). Without plasma treatment of chrysanthemum synseeds, frequency of whole plantlet regeneration varied from 6% to 28% depending on the cultivar. On the other hand, plantlet regeneration from plasma-treated synseeds was 22–49%. The highest treatment effects on whole-plantlet development were recorded for cultivars BC and PP (~370% and ~350%, respectively) in comparison to control synseeds, whereas the lowest values were obtained for the PP cultivar (~160%).

Chrysanthemum plantlets derived from plasma-treated synseeds continued their growth until full physiological maturity and flowering (Figure 4). During further growth of the plantlets under greenhouse conditions, no morphological differences were observed between the plants developed from untreated (Figure 4a,c) and plasma-treated synseeds (Figure 4b,d). Some of the plants originated from synseeds flowered after six months of ex vitro growth (Figure 4f); during the next flowering season, no morphological or color changes in flowers were observed (Figure 4g–i).

### 2.4. Characterization of DBD Plasma Source

**Figure 5:**
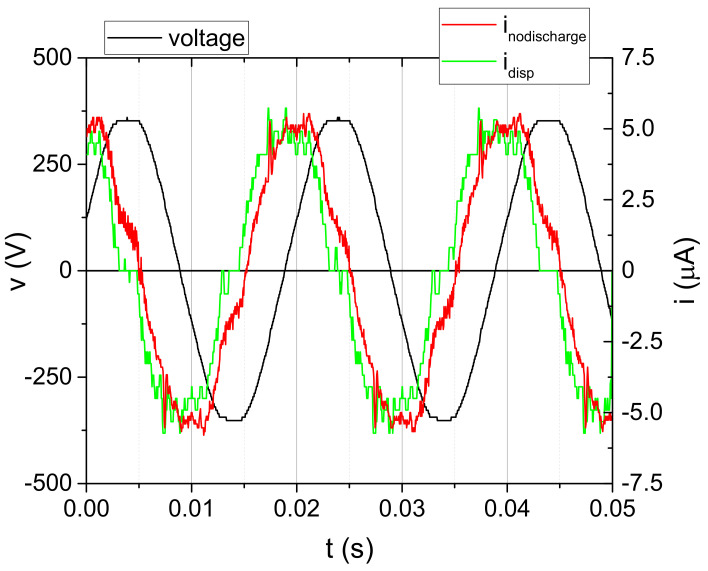
Waveforms of voltage (black line) and current (red line) obtained for d = 5 mm without discharge (input voltage V_input_ = 6 V). Displacement current (green line) is calculated by using voltage signal (Equation (1)).

Detailed plasma diagnostics was carried out prior to the treatments. After assessment of the range of plasma conditions that could be achieved with the DBD source, we selected one condition for the synseed treatment (part 4.3). The SDBD, previously used in treatments of flour [57], was characterized by using commercially available voltage probes. In order to properly assess the discharge current, one needs to subtract the displacement current. The first step was determination of the capacitance of the SDBD when the discharge was not ignited. This capacitance represents the passive capacitance of the plasma system, and it depends mainly on the geometry of the system, so it was determined for several interelectrode distances. The voltage at the powered electrode and current waveforms for one of the cases is represented in Figure 5. The peak-to-peak value of the voltage is 700 V (black line), whereas the peak-to-peak value of the current (red line) is ~12 µA.

The current measured when there is no discharge ignited is only the displacement current. It is represented by:

(1) $$i_{disp}\left(t\right)=C_{p}\frac{dv\left(t\right)}{dt}$$

where *C_p_* represents passive capacitance of the system, and *v*(t) represents instantaneous voltage measured at the powered electrode. As $i_{nodischarge}\left(t\right)=i_{disp}\left(t\right)$, the *C_p_* value calculated as a parameter from Equation (1) for different electrode distances is given in Table 5.

**Figure 6:**
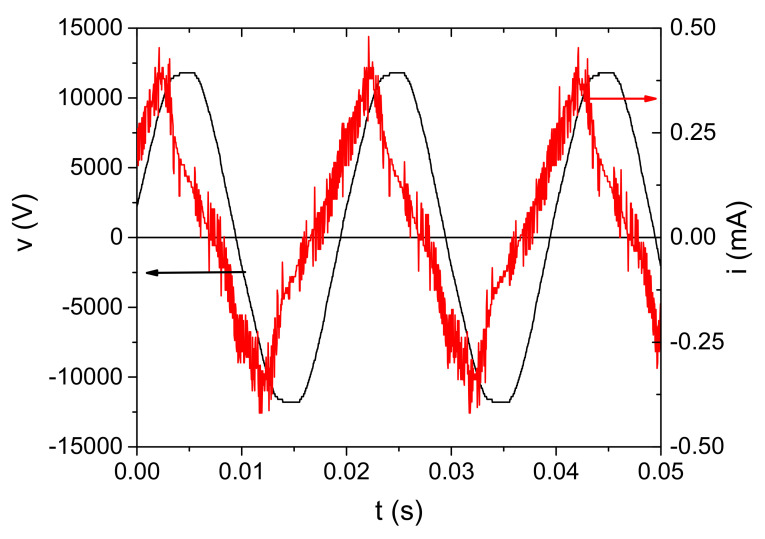
Time dependence of voltage and current signals at V_input_ = 220 V and d = 5 mm for the discharge operating in air.

An example of the voltage and current waveforms with the discharge ignited is shown in Figure 6. The voltage waveform was measured by HV probe at the powered electrode, whereas the current waveform represents the current through discharge. It was obtained by subtraction of the displacement current from the total current measured in the grounded branch of the electrical circuit. Variation of measured voltage and current were below 2% and 4%, respectively. Consequently, the root mean square (RMS) values were calculated with the same uncertainties. We can see that the voltage waveform is a sine function with a dominant first harmonic (50 Hz). The shape of the current waveform reflects a discharge with microfilaments that can be seen through the appearance of current peaks superimposed on the waveform [58,59].

**Figure 7:**
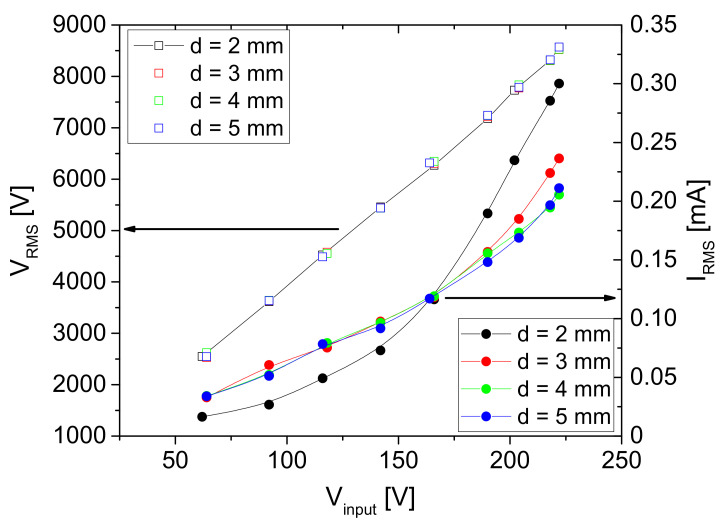
Dependence of the RMS voltage and current values on the input voltage V_input_ for four geometries. The discharge was operating in air.

The dependence of the output RMS voltage measured at the powered electrode on the input voltage is shown in Figure 7, left-hand axis. We can see that the dependence is linear, and it does not change significantly with an increase in the distance between electrodes.

The maximum operating voltage that could be obtained with this power supply system and the plasma source geometries was ~25 kV_peak-to-peak_ (~8560 V_RMS_). The dependence of the measured current on the input voltage is shown in Figure 7 on the right-hand axis. In this case, the dependence was not linear, and it changes with the distance between the powered electrode and the bottom plate, i.e., sample. For all distances presented here, the maximum current was 0.3 m A_RMS_ obtained for d = 2 mm.

**Figure 8:**
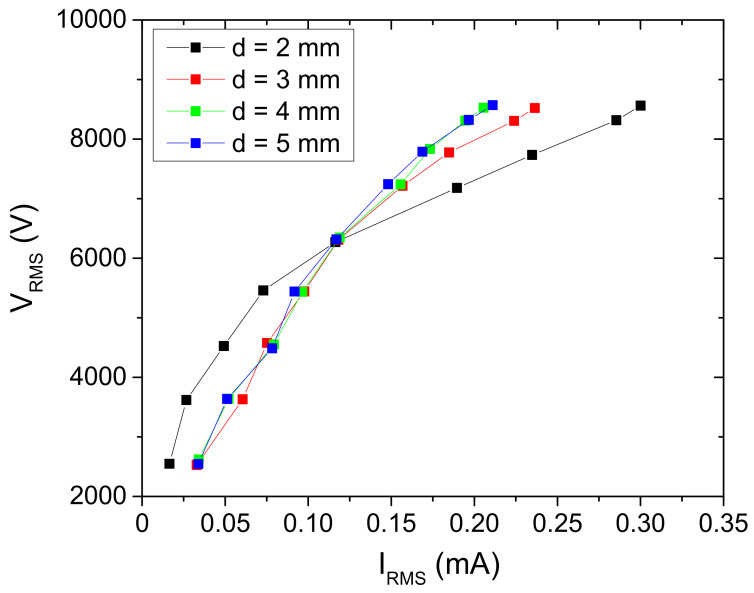
Volt–ampere characteristics of the SDBD obtained for the different distances between the powered electrode and the sample. The discharge was operating in air.

The volt–ampere (V-A) characteristics (Figure 8) show that the system is not linear with constant impedance. The complex impedance changes with an increase in the applied voltage, and the non-linearity is the most pronounced for the distance of 2 mm. This can be explained by the number of microfilaments formed and the effective area that they covered. For the interelectrode distances of d = 3, 4 and 5 mm, the V-A dependence is almost the same up to the applied RMS voltage of 6.5 kV. Increasing of the interelectrode distance reduces the number of microfilaments that occur in one half-period, and the ones that are ignited operate totally with low conduction current.

**Figure 9:**
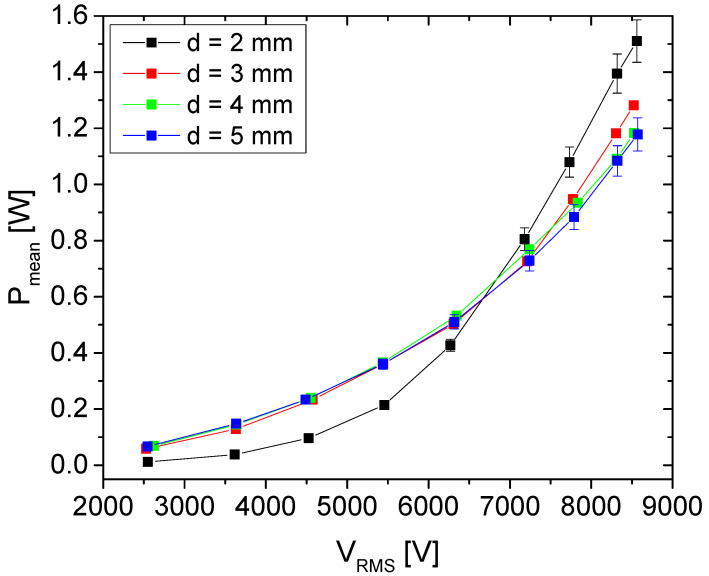
Mean power transmitted to the discharge as a function of the voltage at the powered electrode.

As expected, a similar change can be observed for the power transmitted to the discharge (Figure 9). Power is obtained as a mean value of instantaneous power calculated over several periods. The instantaneous power is obtained by direct multiplication of the waveforms of voltage and discharge current. We can see that the mean power transmitted to the discharge increases with the applied voltage. The maximum power that can be deposited to the discharge is around 1.5 W for the interelectrode distance of 2 mm. For voltages lower than 6.5 kV, the deposited power is similar for distances of d = 3, 4 and 5 mm and lowest for d = 2 mm.

**Figure 10:**
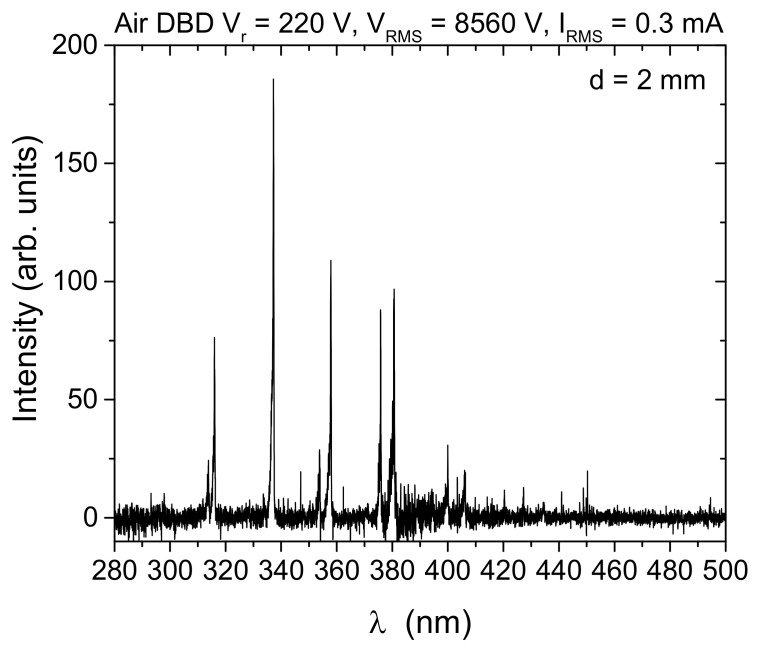
Optical emission spectrum from an air SDBD source obtained from side-on recording of spatially integrated emissions from the whole discharge volume (electrode gap, d = 2 mm). The intensity signal is corrected for spectral efficiency of the optical system.

We recorded the emission spectra of the SDBD plasma source operating in air in the wavelength range from 275 nm to 850 nm. In Figure 10, we present the emission spectrum for d = 2 mm up to 500 nm. Almost all lines visible in the spectrum belong to N_2_ Second Positive System (SPS): 313.67 nm, 315.93 nm, 337.13 nm (head of the band), 350.05 nm, 353.67 nm, 357.69 nm, 364.17 nm, 375.54 nm, 380.49 nm, 399.84 nm, 405.94 nm, 420.05 nm, 426.97 nm, 441.67 nm, 449.02 nm.

## 3. Discussion

First, we will discuss in detail the plasma characteristics used in the study. We used air SDBD because of the plane-parallel geometry, large effective plasma surface and possibility to operate with only air as a feeding gas. When the discharge was ignited, streamers were formed at temporary random points in the form of microfilaments, and they conducted higher discharge current than the rest of the discharge. Because we were not treating plant cells directly but the sodium-alginate-encapsulated plant material, the samples could withstand these local inhomogeneities in the active plasma volume without any damage. Nevertheless, due to the nature of an SDBD source, the total current of filaments was limited, preventing formation of current hot-spots. Another reason for choosing this type of plasma source was its simplicity, both in design and in application. It did not require the addition of feeding gas, and, as an important feature for the future technology, it has a potential for scaling up. Detailed electrical characterization of the SDBD and optical emission spectra was presented where the plasma source, with its plan-parallel geometry, served as a capacitance in the electrical circuit. Regarding this, we used a simple and reliable method to measure the stray capacitance, C_p_, for different distances, *d*, between upper and lower electrode segments. Results showed that with the increase in the distance between the powered electrode and grounded bottom electrode, we had an increase in the system capacitance, which was expected (Table 5). The obtained C_p_ values allowed for determination of the displacement current of each input voltage. The measured current signals included both the displacement and discharge current. Thus, after subtracting the displacement current, all current waveforms represented only current through the discharge. The capacitance determined for each *d* enabled calculation of the discharge current for all possible configurations of the plasma source.

With respect to application, one of the most important macro parameters in plasma treatments is power deposited to the discharge. This macro parameter that can be easily monitored; it reflects the electron density and temperature and, to some extent, through these two parameters, plasma chemistry [60]. P_mean_ (Figure 9) depends on the distance between electrodes, as well as formation of microfilaments. Thus, the highest increase and mean power values were achieved for d = 2 mm. For this distance, we chose to treat the synseeds at a power of 1.1 W. This showed to be the optimal value with respect to the effect on the seeds for the three treatment times that we used.

Apart from power, the feeding gas (in this case air) and humidity can also play an important role in plasma chemistry [61]. Although humidity in the treatment environment was not controlled, all experiments were performed in a room with constant humidity. Additionally, measurements of discharge characterization, as well as all treatments, were repeated several times in order to verify the reproducibility of the measurements. All measurements were performed with and without synseed samples. One way to obtain an insight into the chemical reactions occurring in the discharge is optical emission spectroscopy, as it can show the existence of certain excited species. In our experiment, the spectra were recorded for different RMS voltages and interelectrode distances without samples, but all have the same lines belonging only to the N_2_ Second Positive System, SPS (Figure 10). Absence of the lines of, e.g., NO, OH and atomic oxygen, from the spectrum of an air DBD has been noted before and is related to dominant excitation and quenching reactions that favor N_2_ excitation in filamentary discharges [11,12,61,62]. Moreover, other important reactive species, such as O_3_, do not have emissions in the spectral range investigated. Nevertheless, these kinds of plasma sources generate a large amount of ozone and N_2_O that is important for treatments of alginate surfaces of synseeds [13,14,63,64].

The most important parameters determining the efficiency of the encapsulation and plant recovery of synseeds are survival, regrowth and capability of initial explants for further plantlet growth to complete plantlets [1]. Chrysanthemum synseeds used in this study (plasma-treated and untreated) easily established regrowth with the first leaf emergence after one week of cultivation. The presence of alginate capsules did not inhibit regrowth of chrysanthemum shoots grown on agar medium. In addition, shoots were developed on plant growth regulator free medium without any callus formation, similar to what was reported for chrysanthemum cultivars by the Lady group [19]. Our results are not surprising because regrowth of encapsulated shoot tips of chrysanthemums is possible on plant growth regulation free medium [19]. In the case of encapsulation of nodal segments, the addition of a small amount of IAA in the encapsulation complex is necessary and obligatory for better regrowth of shoots from synseeds [29]. When synseeds were grown on VLM only, plasma-treated chrysanthemum synseeds continued their regrowth and formed well-developed microshoots. It should be mentioned that microshoots established on VLM were smaller than those developed on standard solid agar medium, which could be related to accessibility of nutrients in this type of medium.

Data about application of synseed technology for short- and long-term storage in vitro or easy transport of valuable genetic resources are available [65,66], whereas data regarding synseed manipulations for ex vitro growth are quite scarce [67,68]. In chrysanthemum, plantlet development from synseeds formed in vitro and sowing ex vitro was successfully achieved from double-layered synseeds [29]. In the current work, we planted untreated and plasma-treated simple, one-layer chrysanthemum synseeds directly in soil substrate. After three weeks of ex vitro cultivation of chrysanthemum synseeds, two-fold higher shoot development was observed in the case of plasma-treated chrysanthemum synseeds in comparison to untreated synseeds. Additionally, after six weeks of growth under ex vitro conditions, plasma-treated chrysanthemum synseeds showed significantly higher plantlet conversion compared to the untreated control. The enhanced survival, regrowth and further complete plantlet formation of plasma-treated chrysanthemum synseeds shown in our work might be explained, besides by antimicrobial effect, by the prolonged effects of chemical changes in the alginate gels after plasma treatment and their antimicrobial properties. Similar effects were reported for plasma treatment of alginate wound dressings [12]. Plasma treatment of alginate gels inactivated bacterial and fungal infection for a month, which was a long enough period for successful shoot development and complete conversion of synseeds to plantlets. On the other hand, continued growth of untreated chrysanthemum synseeds was significantly reduced due to contamination and lack of adventitious root formation for other plant species [9].

We found that the plasma treatment significantly enhanced synseed germination and complete plantlet development for all investigated chrysanthemum cultivars. The response to plasma treatment of chrysanthemum synseeds was cultivar-dependent. Observed differences could be attributed to the fact that different chrysanthemum cultivars have distinct nutritional requirements, as was reported earlier for other chrysanthemum cultivars [19,30]. According to our results, no morphological disorders were noticed among plantlets derived from untreated and plasma-treated synseeds. The absence of any morphological or flower color alterations in chrysanthemum plants may be explained as a consequence of the regeneration protocol used in this study. First, we used stock shoot cultures derived from one mother plant. In addition, initiation of shoot regeneration was mainly achieved by direct shoot induction on the initial explant, avoiding a callus phase and further shoot multiplication by axillary meristem activation, which minimized possibilities for genetic changes due to somaclonal variations [16,17]. According to available data, this research represents first data about complete chrysanthemum plantlet regeneration from synseeds to flowering plants.

During direct sowing of synseeds, contamination by microorganisms is one of the major hurdles for the commercialization of encapsulation technology for many plant species [9]. Besides that, one of the main limiting factors for plantlet conversion is low-nutrient availability due to inhibition of root growth. Numerous factors are involved in this process, such as poor rooting ability and survival due to the lack of nutrients and oxygen supply. Organic nutrients released by the beads are mainly responsible for severe contamination of synseeds [29,30]. Unfortunately, the depletion of nutritional compounds in beads may cause lower shoot regrowth or complete growth inhibition [28,29,30]. To date, there are two strategies to overcome this problem in chrysanthemum synseeds. The first strategy is to use double-layered synseeds to restrict contamination, where the second layer is formed by Ca-alginate made with water [29]. A recently reported strategy for both production and sowing of chrysanthemum synseeds in non-aseptic conditions proposes eliminating all carbon sources and organic additives both inside and outside the synseeds [30]. The reported strategy might be promising, but it was applied to one cultivar only, and it is questionable whether it is applicable to other cultivars. Therefore, it is necessary to build up a system that lowers contamination and keeps a nutrient reservoir within the encapsulated plant tissue, which is necessary for successful rooting. Considering the results of the present study, this problem may be successfully solved by plasma treatment of chrysanthemum synseeds before sowing.

There are many research data that demonstrate that cold plasma has a potent general antimicrobial effect through its generation of free radicals, reactive oxygen species (ROS) and reactive nitrogen species, such as hydrogen peroxide, superoxide, singlet oxygen, nitric oxide and ammonia [42]. The generated reactive species or their products are responsible for the antimicrobial effect and, in certain situations, have proven to be non-toxic to eukaryotic cells [69]. The chemical changes produced in the gel are relatively stable, and the anti-microbial properties of such a gel may last for close to one month, as reported after plasma treatment of alginate wound dressings [13]. The treated alginate gels inactivated all of the Gram-negative, Gram-positive and fungal pathogens by generated ROS inside bacterial cells, leading to their rapid death or triggering programmed cell death exhibiting characteristic features of apoptosis [12]. According to our results, we can conclude that treatment with non-thermal plasma generates chemical and physical responses in an alginate gel, producing changes that implicate not only biocidal effects but possibly growth-promoting effects.

## 4. Materials and Methods

### 4.1. Atmospheric Pressure Plasma Source

**Figure 11:**
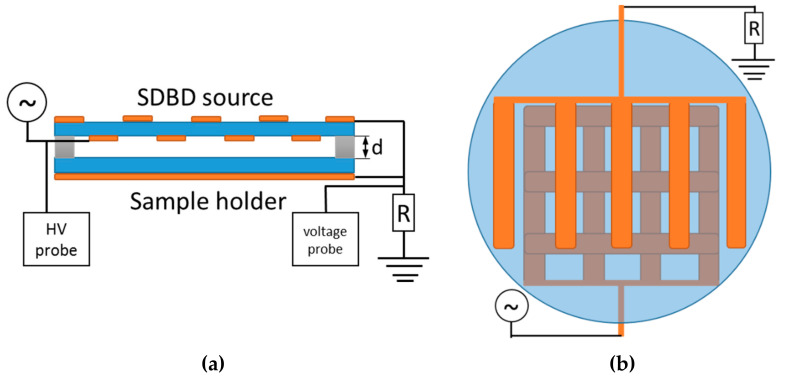
SDBD source schematics: (**a**) side view of the setup and schematics of electrical circuit; (**b**) top view of an upper electrode part. Dimensions of the source are given in the text.

In the experiments, we used a circular surface dielectric barrier discharge (SDBD) source with an outer diameter of 90 mm. Side- and top-view schematics of the source are shown in Figure 11.

The source consists of the two plane-parallel glass plates (2 mm thickness). The lower glass plate is positioned on a stainless-steel plate and serves as a sample holder. The upper glass plate is covered with conductive strips made of 5 mm wide copper tape. The strips are placed on both sides of the glass dielectric (Figure 11a). On the top side, they form a comb-like structure with gaps between the strips of 12 mm (Figure 11b). On the bottom side, the conductive strips form a grid structure making 12 mm side squares. The strip structures on opposite sides of the glass plate are shifted in such way that longer sides of the conducive strips are not overlapping (Figure 11b). The copper strips fixed to the upper part were grounded, whereas the strips on the bottom part of the top glass plate were powered with 50 Hz high-voltage (HV) sine signal. The outer edges of both powered and grounded electrode structures on the top part form a rectangle. Along the sides of this rectangle, plastic spacers were placed, keeping fixed distance in between the glass plates and bordering the active plasma volume of the SDBD source. Characterization of the device was performed by using spacers with thicknesses (d) of 2, 3, 4 and 5 mm.

The HV signal at the powered electrode was supplied by a homemade HV transformer. The input voltage (V_input_) to the HV transformer was provided by a variable voltage regulator connected to the electrical power grid. The HV signal was monitored by a high-voltage probe (Tektronix 6015A, North Star High Voltage, Beaverton, OR, USA) connected to the circuit close to the powered electrode. Grounded electrodes on the top and bottom part of the source were connected to the same grounding point. In this grounded line, the probe (Agilent 10076A, Agilent Technologies, Beijing, China) allowed tracing of the voltage drop on the R = 15 kΩ resistor, thus monitoring of the total current in the discharge. Electrical signals were recorded by oscilloscope (Agilent DSO6052A, Agilent Technologies, Beijing, China) and saved on a computer for further processing.

### 4.2. Synthetic Seed Production

**Figure 12:**
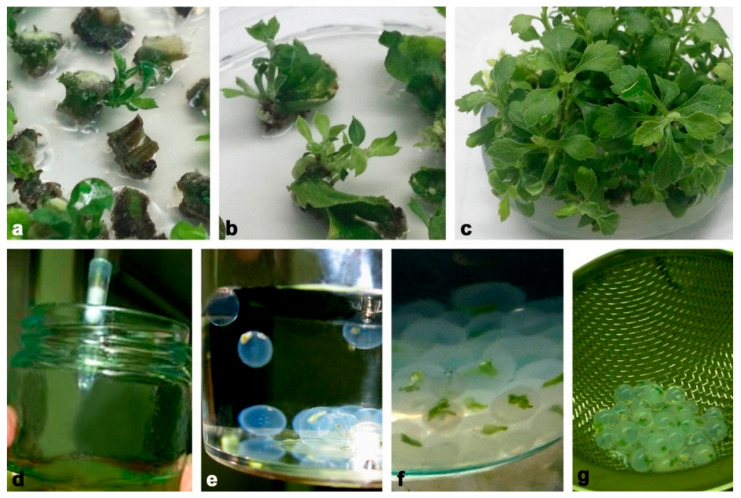
Chrysanthemum synthetic seed production. (**a**,**b**) Initiation of shoot regeneration in steam segment of cv. PC (**a**) and leaf culture of cv. BL (**b**); (**c**) stock shoot cultures used as primary explants for synthetic seed production; (**d**) sucking of shoot tips with sodium alginate; (**e**) formation of alginate beads in complex solution consisting of 100 mM CaCl_2_ × 2H_2_O; (**f**) well-formed alginate beads after 20 min in complex solution; (**g**) washed synseeds ready for treatment.

We used four chrysanthemum cultivars as starting plant material for the experiments: Brandsound Liliac (BL), Précocita Carna (PC), Précocita Parme (PP) and Queens (Q). Shoot cultures were established from one branch of the mother plant of each cultivar. Nodal and internodal stem (Figure 12a) and leaf segments (Figure 12b) of the mother plant were, after surface sterilization, grown on Murashige and Skoog mineral solution and vitamins [70] solidified with 7% agar, with the addition of 3% sucrose and 100 mg/L myo-inositol (MS medium) supplemented with α-naphthylacetic acid (0.1 mg/L NAA, Sigma-Aldrich, St. Luis, MO, USA) and 1.0 mg/L 6-benzylpurine (BAP, Sigma-Aldrich, St. Luis, MO, USA) for shoot induction. Initially regenerated shoots were further subcultured on the same medium, and stable stock cultures of chrysanthemum cultivars were established and subcultured in 4-week intervals (Figure 12c).

For production of synthetic seeds, we used axillary shoots developed from nodal stem segments (1 cm) grown for 3 weeks on plant regulator free MS medium described above. After 3 weeks of growth, shoot tips (2–3 mm) were excised from same-sized axillary shoots (2 cm) and washed for 20 min in MS liquid medium without CaCl_2_ × 2H_2_O. Encapsulation was performed according to the following procedure: the explants were plunged into a solution of sodium alginate (2.5%, *w*/*v*, medium viscosity, Carlo Erba, Carnadero, Italy) made with MS liquid medium without CaCl_2_ × 2H_2_O. Subsequently, droplets of the alginate solution containing one shoot tip were sucked into pipettes with shortened sterile plastic tips (Figure 12d) and dropped into a complex solution (Figure 12e) consisting of 100 mMCaCl_2_ × 2H_2_O (Sigma-Aldrich, St. Louis, MO, USA). Formed alginate beads (5–6 mm in diameter) were maintained for 20 min in this solution with continuous slow agitation (Figure 12f). The encapsulated shoot tips (synseeds) were retrieved and rinsed three times in sterile distilled water in order to remove traces of CaCl_2_ × 2H_2_O (Figure 12g). Finally, water was decanted, and synseeds were placed on sterile filter paper for a few minutes (Figure 1a).

All nutritional media were adjusted to pH 5.8 before sterilization. All media, sodium alginate and complexing solution were sterilized in an autoclave for 20 min at 114 °C. In vitro cultures were grown in a growth room at 23 ± 2 °C, with a photoperiod of 16 h day/8 h night.

### 4.3. Plasma Treatment of Synthetic Seeds

Chrysanthemum synseeds (Figure 1a) of the same size (5–6 mm in diameter) were exposed to an air SDBD. Twenty-five synseeds were gently placed onto a sterile glass Petri dish (diameter, 90 mm) and then placed below the powered electrode in unsterile conditions and exposed to the plasma all at the same time. The distance between the powered electrode and the synseed surface was ~2 mm. Due to the fact that seeds are ball-shaped and vary slightly in size, the distance could only be estimated. The treatment times were 1 min, 5 min and 10 min. The applied HV in all treatments was V_peak-to-peak_ = 22 kV (V_RMS_ = 7.8 kV), which corresponded to a mean power of 1.1 W.

### 4.4. Sowing of Synthetic Seeds

Untreated and plasma-treated synseeds were grown on three different sowing substrates as follows: (i) sterile agar MS medium (AM), grown under in vitro conditions; (ii) sterilized vermiculite + liquid MS medium (VLM), grown under in vitro conditions; (iii) direct sowing into unsterilized soil substrate, grown under ex vitro conditions (greenhouse).

For the in vitro experiment, untreated (control) and cold plasma-treated chrysanthemum synseeds were grown on solid plant regulator free MS medium (30 mL) filled in baby jars. Similarly, untreated and cold plasma-treated synseeds (5 per baby jar) were grown in baby jars (5) filled with 10 g vermiculite and moisture with 10 mL liquid plant regulator free MS medium. Baby jars filled with 10 g of vermiculite were sterilized in an autoclave for 20 min at 114 °C. In vitro cultures with synseeds grown on AM and VLM were grown in a growth room at 23 ± 2 °C, with a photoperiod of 16 h day/8 h night. Germination of the synseeds under in vitro conditions was estimated as a frequencies of shoot regrowth from alginate beads at two steps: leaf emergence after one week and full shoot development after four weeks of cultivation.

For the ex vitro experiment, untreated (control) and cold plasma-treated synseeds were sowed directly in plastic containers with 18 places (3 × 6) filled with commercial substrate (Floradur^®^ Seed, Floradur, Oldenbürg, Germany) during April and May. Two synthetic seeds were placed on the top of the substrate, one in each place inside a plastic container. All containers were covered with transparent foil during the first four weeks of growing. The synseeds were sprayed weekly with unsterile MS mineral solution. Germination of synseeds under ex vitro conditions was estimated as a frequencies of leaf emergence from alginate beads after one week of cultivation, full shoot development was assigned as complete shoot regrowth from initial synseed after three weeks of cultivation and whole-plantlet formation was recorded when germinated synseeds developed roots after 6 weeks of culture. Total deterioration (%) was evaluated as a percentage of seeds that failed to regrow (germinate) and regenerate to plantlets with respect to the total number of planted synseeds. After twelve weeks of growth in containers, each synseed plantlet was potted in individual pots filled with a mix of peat and perlite (3:1) and grown under greenhouse conditions until flowering. Experiments with plasma treatment were repeated two to four times, and 15–25 synthetic synseeds were used per treatment depending on chrysanthemum cultivar. All data were subjected to statistical analysis using STATISTICA and analysis of variance (ANOVA) using least significance (LSD) tests.

## 5. Conclusions

Synseed technology may be a useful technique for a propagation systems in terms of fast reproduction of seedless plants, preservation of the genetic uniformity of plants, straight delivery to the field and, last but not least, low cost. The difficulties of sowing artificial seeds directly in soil or on commercial substrates under non-sterile conditions are considered to be one of the main limitations for the practical use of this technique. In this paper, we presented, in detail, the results of the electrical characterization of an SDBD that operates in air and its possible application in synthetic seed technology. According to our results, implementation of the SDBD plasma treatment before sowing represents a promising strategy for future investigations and sustainable use of cold plasma in synseed biotechnology. Plasma-treated chrysanthemum synseeds showed a better survival rate and overall plantlet growth under greenhouse conditions in comparison to untreated synseeds.

In conclusion, to the best of our knowledge, this is the first report about the use of SDBD plasma for seed germination of synthetic seeds under aseptic or non-aseptic conditions. This study demonstrated a highly effective strategy for direct conversion of synseeds into entire plantlets by using plasma pre-conversion treatment. This treatment reduced contamination and displayed a considerable ex vitro ability to convert clonally identical chrysanthemum plants.

## Figures and Tables

**Table 1:** Application of synseed technology for storage and propagation of chrysanthemum cultivars.

Cultivar	Plant Material	Beads	Sowing	Germination (%)	Flowering	References
Clone ‘PS 27’	Nodal segments (in vitro)	Monolayered (3% Na-alginate + 0.1 mg/L IAA)	Sterile, water–sand	50	no	[29]
		Double-layered (beads: 3% Na alginate, second layer: water)	Non-sterile, water–perlite	45	no	
Lady group	Shoot tips (in vitro)	Monolayered (3% Na-alginate)	Sterile, agar	52	no	[19]
cv. ‘Royal Purple’	Shoot tips (ex vitro)	Monolayered (2.5% Na-alginate, sucrose, vitamin free)	Non-sterile, vermiculite	34	no	[30]

**Table 2:** The effect of plasma treatment on germination of chrysanthemum synseeds grown in vitro on vermiculite + liquid medium.

Plasma Treatment (min)	Leaf Emergence (%) *	Shoot Regrowth (%) **
	1 Week	4 Weeks
0	20 ± 10 ^a^ ***	0 ± 0 ^a^
1	47 ± 13 ^a,b^	40 ± 13 ^b^
5	67 ± 12 ^b^	47 ±13 ^b^
10	67 ± 13 ^b^	33 ± 12 ^b^

* Leaf emergence is evaluated as first sign of shoot appearance out of alginate beads after one week of growth; ** shoot regrowth was evaluated as fully developed shoot out of beads after four weeks of growth. *** values represent mean ± standard error. The data signed with different letter within the same column are significantly different according to Fisher’s LSD test.

**Table 3:** The effect of plasma treatment on germination of chrysanthemum synseeds grown ex vitro.

Plasma Treatment (min)	Synseed Germination		
	Leaf Emergence * (%)	Shoot Regrowth * (%)	Plantlet * (%)
0	60 ± 7 ^a^ **	33 ± 7 ^a^	17 ± 5 ^a^
10	66 ± 5 ^a^	60 ± 5 ^b^	41 ± 5 ^b^

* Leaf emergence is evaluated as first sign of shoot appearance out of alginate beads one week after sowing; shoot regrowth was evaluated as a fully developed shoot out of the bead three weeks after sowing; plantlet development was recorded as a fully developed plant with well-developed shoot and roots. ** Values represent mean ± standard error. The data signed with a different letter within the same column are significantly different according to Fisher’s LSD test.

**Table 4:** The effect of plasma treatment on synseed germination and plantlet development of different chrysanthemum cultivars.

Plasma Treatment (min)	Plantlet (%)			
	BC *	Q *	PC *	PP *
0	6 ± 1 ^a^ **	17 ± 2 ^a^	28 ± 1 ^a^	14 ± 1 ^a^
10	22 ± 6 ^b^	40 ± 3 ^b^	44 ± 2 ^b^	49 ± 3 ^b^
Increment (%)	~370	~230	~160	~350

* Chrysanthemum cultivars: Brandsound Liliac (BC), Queens (Q), Précocita Carna (PC) and Précocita Parme (PP); ** values represent mean ± standard error. The data signed with different letters within the same column are significantly different according to Fisher’s LSD test.

**Table 5:** Values of stray capacitance in the SDBD system.

Electrode Distance-d [mm]	Stray Capacitance—*Cp* [pF]
2	20
3	35
4	41
5	45

## Data Availability

All data are shown in the manuscript, and raw data are available from corresponding authors upon reasonable request.

## Supplementary Materials

The following are available online at https://www.mdpi.com/article/10.3390/plants11070907/s1, Figure S1: Shoot multiplication index in a control group (plasma −) and with plasma treated synseeds (plasma +).
