# Supplementary material for: Treatment of Chrysanthemum Synthetic Seeds by Air SDBD Plasma
Source: Plants (Basel). 2022 Mar 29;11(7):907. doi: 10.3390/plants11070907 (PMC9003063; doi:10.3390/plants11070907)
Supplement: Supplementary file 1 [file plants-11-00907-s001.zip › 1.pdf]

Supplementary data S1- Treatment of chrysanthemum synthetic seeds by air SDBD plasma

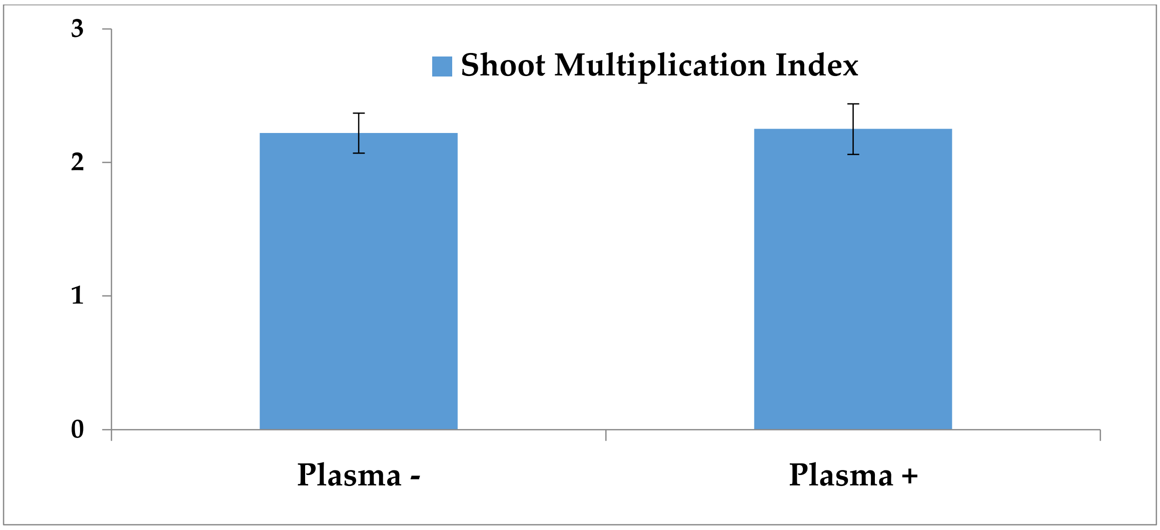

Figure S1. Shoot multiplication index in a control untreated (plasma -) and plasma-treated synseeds (plasma +).
